# Oral Surgery

**Published:** 1874-01

**Authors:** 


### ARTICLE III.

### *Oral Surgery.*

The *Philadelphia Medical Times*, in its issue of November 1st, taking the editorial of the October number of the *Dental Cosmos* for a text, discusses the question whether

Oral Surgery is sufficiently distinct and of sufficient magnitude to be worthy of rank as a specialty of medicine. Admitting specialism to be a necessary outgrowth of the extension of medical science and art, the writer recognizes the danger of carrying it too far,—of dividing up too finely. He then considers the true position of dentistry and of dentists, claiming that the great bulk of the work to be done is purely *mechanical*, and therefore the mass of the profession must spend their lives in a monotonous round of purely mechanical labor, in which mechanical and artistic skill along with personal qualities must be the sole guarantees of success; that the higher education and wider culture of the physician is in no sense a necessity to such success; and that dentists as a class have no claims to be recognized as representatives of a branch of the medical profession. He assumes that, because a man may be an excellent doctor without being a dentist, therefore he may be an excellent dentist without being a doctor, and argues that as cancer of the jaw is essentially the same as cancer of the rib, vertebra, or tibia, therefore there is no necessity for oral surgery being a specialty. The writer, however, practically refutes all that he had written in the final admission that he "*would like to see dental schools attached to our medical colleges, and opportunity afforded our medical students to learn something of diseases of the teeth, or even, if they like, to become practical dentists.*" Diseases, forsooth! why, we have just been told that the work of the dentist is purely *mechanical*. Now it seems there are *diseases* of the mouth, which it is desirable medical students should learn something about. That doctors *should* learn something of dental specialism, and that dentists *should* learn more of physiology, pathology and therapeutics, is just exactly that for which we have been contending. That there is abundant room for improvement in both directions, we are well assured; but especially sure that no branch of medicine or surgery is so little comprehended by the general practitioner as that which is assigned to the province of the educational dental specialist. If in any complex dental lesion, the "mechanical labor" of the dentist, is not sufficient, heaven help the patient who expects to supplement it with the therapeutices of the average family physician! Or, if such complex lesion should first present to the family physician, he would be in an awkward position if compelled to commend him to the care of one whom he had pronounced in no sense entitled to be recognized as a representative of a branch of our profession.

The one word *disease* is a solution of the whole problem; for without a knowledge of anatomy and physiology, how can pathological conditions be intelligently diagnosed; a clear insight obtained into the causes which interfere with normal results or the morbid changes induced by disturbance of physiological processes; and of what use would this knowledge be to one ignorant of therapeutics?

We claim that a hiatus has existed between general medicine and surgery and dentistry; a hiatus not likely to be bridged by general practitioners; that every day there is needless suffering from the lack on their part of the special information which belongs to the province of the dentist. And while patients may not expect from them the manipulative skill which comes only through practice, they are justified in expecting at least an intelligent appreciation of their difficulties. This they are not likely to have while the general practitioner so far fails to recognize dental pathology,—its regional and systemic relations,—as to designate its treatment as "purely mechanical."

The critic of the *Times* finds further argument against oral surgery as a specialty, because "it has no natural boundaries to limit it;" for, says he, "this very day, chancing to be at the clinic" of the lecturer upon oral surgery at the University of Pennsylvania, we saw present three cases, two of which were tumors on the head. What does this prove, except the advantage of such a general medical education as will qualify for the practice of *any* branch? One who makes oral surgery a specialty, need not, if he elect otherwise, decline the treatment of any case. The argument is only an indorsement of the higher education and wider culture for which we contend.

But let us reverse the picture. "Chancing, this very day," to step in at the house of a medical friend, a general practitioner, we found his wife suffering from an atrocious alveolar abscess, of the pathology and therepeutics of which the doctor was as innocent as a child, and had not recognized the imminent risk of an external disfiguring scar or fistula. This is not an extreme or isolated case. Doctors are proverbially ignorant of the proper treatment of the simplest dental lesions, and we doubt if one in ten can tell the period and order of eruption of the deciduous teeth, or explain the relation of the first molar to the temporary and permanent dentitions. If it be replied that such knowledge ought not to be demanded of the general physician, a fitting answer would be the fable of the dog in the manger. That doctors as a class are not familiar with dental lesions will not be disputed, and the effort to belittle the practice of those to whom they themselves apply when suffering in their own persons from such conditions, is, to say the least, in bad taste.

While therefore we urge a wider range of knowledge upon the dentist, we hail the suggestion that our "medical students be afforded opportunity to learn something of the diseases of the teeth," with great satisfaction; that thus the minds of both may be disabused of the false conception that either may afford to ignore that which belongs to the circle of the other. It is as impossible that any one man should be master of medical science in its details, as it is for him to attain the best possible results in the smallest specialty except by an appreciation of general principles, which underlie equally the practice of medicine and of dentistry.

Dentists are either artisans or medical specialists. If artisans only, they surely are not entitled to recognition as representatives of a branch of the medical profession; and no mechanical skill, however scientific, no artistic culture, however perfect, should be expected to rank for more than these. If dentistry mean only mechanical and artistic ability, these can be acquired without colleges, and employed without degrees; but if its claim to recognition as a medical specialty is worth *anything*, it is worth *all* it represents.

In the article reviewed, we had distinctly affirmed that the attempt to appropriate the honors of a learned profession on mechanical and artistic ability was simply to court ridicule. With so much of the argument, therefore, as is based on the assertion that knowing how to plug a difficult molar or counterfeit a lost incisor does not entitle to recognition as a specialty of medicine, we heartily agree. We not only admit, but have contended, that mere mechanical and artistic ability does not entitle its possessor to the doctorate, but we deny that a man may be an excellent dentist without being a doctor; we deny that the higher education and wider culture of the physician are in no sense a necessity to the practitioner of dentistry, and demand that he should possess the higher education and wider culture, or lacking them should content himself without a title.

The question is not what dentistry has been, but what oral surgery should be; not what are the qualifications of the majority of those now practicing it, but what is its legitimate province, and what the requirements for its intelligent practice. We see no force in the assumption that because the great bulk of the profession have heretofore spent their lives in a monotonous round of purely mechanical labor, therefore they must continue to do so in the future. We claim that the circle of physiological and pathological sympathies existing between the mouth and every portion of the economy demand first a general medical education, and then special training, that the highest results in treatment may be secured. And as each year witnesses a more thorough educational training, so will the field inevitably widen, until the function of the dentist will be merged into a practice of which that of to-day is but a feeble indication. Who doubts this, let him look at the beginnings of ophthalmology as compared with the practice of that specialty in modern times. Who could have guessed its present proportions?—nay, who can yet tell its future triumphs?

The mistake of our reviewer consists in ignoring the variety and importance of dental lesions, and in assuming that they can be successfully combated by mechanical labor, —a mistake he would not have been liable to, had he enjoyed the opportunities which he would like to see offered to our medical students. As to the necessity for special schools for any department of medicine, that is a question open for discussion; but a complete medical education having been obtained as a foundation, on this basis the study and practice of any specialty is entitled to the respect and confidence not only ot the community, but of all who base their claim to recognition on a like scientific preparation. We claim, therefore, that a dentist having a common education with the general surgeon is entitled to common honor and common privileges with him, and hope to see the day when distinctions because of different fields of labor will be done away.—*Editorial of Dental Cosmos.*

